# A Design of Experiment (DoE) Approach to Evaluate the Recyclability of a Polypropylene Copolymer in Medical Technology Under the Aspect of Additive Composition

**DOI:** 10.3390/polym18010083

**Published:** 2025-12-27

**Authors:** Nele Espelage, Markus Lothar Susoff, Cathrin Schröder, Peter Blömer, Svea Petersen

**Affiliations:** 1 Laboratory of Polymer Engineering, University of Applied Sciences Osnabrück, Albrechtstraße 30, 49076 Osnabrück, Germany; 2 Center for Materials and Technology, Private University of Applied Sciences for Business and Technology, Am Campus 3, 49356 Diepholz, Germany

**Keywords:** design of experiment, recycling, sterilization, polypropylene

## Abstract

This study evaluates the influence of repeated processing, γ-irradiation (25 kGy), and additive formulation including Irgafos 168 (I168), Tinuvin 622 (T622), and Calcium Stearate (CaSt) on a polypropylene copolymer (PP-C). Motivated by medical technology applications, the study assessed effects on optical properties, yellowing, crystallization, mechanical performance, and viscosity using a full factorial design of experiments (DoE). Results showed γ-irradiation had the most significant impact, especially on zero-shear viscosity, which decreased by 84% after the first irradiation. The Yellowness Index (YI) changed measurably, but discoloration remained imperceptible. Crystallization temperature was influenced mainly by additive interactions, while specific enthalpy was affected by processing and γ-irradiation. Elongation at break and tensile strength were predominantly influenced by γ-irradiation, with elongation at break being a sensitive indicator of degradation. Zero-shear viscosity, correlating with molecular weight, was mainly controlled by γ-irradiation, indicating chain scission without critical embrittlement. Overall, γ-irradiation exerted a stronger effect than processing or additive formulation. Zero-shear viscosity proved a reliable measure of degradation, while elongation at break offered complementary insights. Despite significant viscosity reduction, mechanical properties remained high, confirming the material’s suitability for its intended applications.

## 1. Introduction

Single-use plastics have sustainably improved the healthcare sector by maintaining sterility and functionality. In addition, the variety of plastics and their adaptability enable a wide range of possible applications. Nevertheless, the global goal is to reduce plastic waste, but until now, hospital waste has been disposed of by incineration due to the risk of contamination. For this reason, sustainability in the handling of plastic waste from medical technology has gained more attention in recent years [1].

Most plastic products for the medical sector are developed primarily in terms of performance and cost efficiency. Due to the high requirements in the healthcare sector, less attention was paid to waste management and recycling [2]. The healthcare sector is responsible for 4.6% of global greenhouse gas emissions, partly due to the supply chain and more resource-intensive facilities [3]. Surgical healthcare contributes significantly to waste generation in the healthcare sector. Every year, 315 million operations are performed worldwide, and the waste generated accounts for 70% of hospital waste. For example, M. Pegg et al. analyzed the waste generated during primary hip surgery, of which 30% consists of plastics and disposable items. It was found that an estimated 15% of the waste generated contains clean recyclable waste materials, including plastics [4].

If recycling is to make a future difference in medical technology, various factors must be considered. Among other things, the influence of repeated processing and sterilization on the material properties must be considered. A study showed repeated processing of a polypropylene (PP) ethylene block copolymer provides a decrease in complex viscosity, while the crystallinity of the PP fraction remains unchanged [5]. The multiple processing of PP, moreover, results in reduced relative elongation at break or increased yellowing [5,6]. The sterilization method is particularly important. For instance, Kremser et al. compared sterilization using γ- irradiation and eBeam at a dose of 45 kGy. This study showed that the damaging effect of γ- irradiation on a commercial radiation-resistant PP is greater than the effect of eBeam [7,8]. The combination of eBeam and γ-radiation has hardly been investigated to this point.

The influence of repeated processing and ionizing irradiation is dependent on the material composition. To mitigate the dominant influence of irradiation, numerous studies have investigated the use of additives as well as polymer selection and modification strategies. To date, these approaches have only been able to reduce, but not suppress, radiation-induced degradation. Complete suppression is not achievable at higher irradiation doses. T. Kremser reported that the radiation stability of polypropylene improves with increasing molar mass and decreasing crystallinity [8]. To mimic typical use, typical additives were selected and tested in every combination. The formulation of the polypropylene compound contained a hindered amine light stabilizer (HALS), Tinuvin 622, the secondary antioxidant Irgafos 168, and the acid scavenger calcium stearate. The effects of the individual additives have already been investigated in a wide variety of studies. However, the combination and interactions between the additives have not yet been the focus of attention. Some studies on additives are listed in the following section. For example, I. Hussain and H. H. Redhwi investigated the stabilizing effect of various hindered amine light stabilizers (HALS) by outdoor weathering influenced by light. The presence of different HALS delays the formation of oxidation products [9]. A study by M. Hamskog et al. investigates the difference between two base stabilization systems, which use phenolic and/or phosphitic antioxidants to slow down the material degradation of PP [6]. Phenolic antioxidants belong to the group of primary antioxidants, which are mainly used to suppress oxidation during processing. Associated color changes of these stabilized products due to aging or processing influences can be attributed to the structural changes of the stabilizer itself. Alternatively, in order to suppress color changes, secondary antioxidants are additionally applied [10]. This combination of phenolic primary antioxidants with phosphite secondary antioxidants is more resistant to degradation reactions during processing and irradiation [11]. Since yellowing is not tolerated for most medical products, the present study will not initially consider phenolic antioxidants.

It is becoming apparent that many different factors need to be considered when evaluating the recyclability of plastics within the medical sector. This study focuses on a PP copolymer. With a total share of 18.9% of global plastics production, PP offers a great deal of leverage and is used as standard in medical technology as a packaging material, but also as a functional product [12]. In order to evaluate the influence and interactions of multiple factors on selected material properties, a full-factorial statistical test plan from the Design of Experiments (DoE) approach was chosen. This method is used for the efficient planning and evaluation of test plans under the influence of many influencing variables [13]. The selected factors considered in the subsequent DoE are, in addition to the influence of repeated processing and sterilization, the influence of the presence of various additives. The chosen sterilization method is γ-irradiation, as it is a significant sterilization method with a market share of 40% [7]. The responses YI, crystallization temperature, elongation at break and zero shear viscosity were evaluated to assess the influencing factors. The YI is used to evaluate color changes. The crystallization temperature, as well as the zero viscosity, are good parameters for evaluating molecular degradation. Elongation at break is a sensitive parameter for evaluating the influence of molecular degradation on mechanical properties [7,14].

## 2. Experimental

### 2.1. Materials

Isotactic polypropylene copolymer (PP-C) containing <10 mol% ethylene units was supplied by Borealis Polymere GmbH (Burghausen, Germany). The additive T622, systematically named poly(4-hydroxy-2,2,6,6-tetramethyl-1-piperidine ethanol-alt-1,4-butanedioic acid), and the Additive I168, systematically named tris(2,4-di-tert.-butylphenyl)phosphite, were provided by BASF Schweiz AG (Kaisten, Switzerland). CaSt, [CH_3_(CH_2_)_16_COO]_2_Ca, was furthermore sourced from Borealis Polymere GmbH (Burghausen, Germany). Methanol, acetonitrile and toluene, each with a purity of 99.8%, and ethyl acetate with a purity of 99.5%, were procured from Fisher Scientific GmbH (Schwerte, Germany). Ethylenediaminetetraacetic acid disodium salt dihydrate (Titriplex III), indicator buffer tablets, 10 M nitric acid, and ammonium hydroxide solution (≥25%) were supplied by Merck KGaA (Darmstadt, Germany). Potassium hydroxide (KOH) was obtained from Fluka AG (Buchs, Switzerland), while 1-(2-hydroxyethyl)-2,2,6,6-tetramethylpiperidin-4-ol (Diol 622) was supplied by abcr GmbH (Karlsruhe, Germany).

### 2.2. Preparation of Test Formulations

Test formulations were prepared using a ZE28 x 44D–BP-UG extruder (KraussMaffei Group GmbH, Parsdorf, Germany). The extrusion setup corresponded to the ZE28 BP standard (KraussMaffei Group GmbH, Parsdorf, Germany) with a screw length of 44.00 D = 1232.00 mm. To produce the test formulations, the additives T622, I168 and CaSt were gravimetrically dosed according to Table 1. The additives at the specified concentrations were subsequently used in all possible combinations to prepare the compounds. Based on a design of experiments (DoE) approach in which the additives I168, T622, and CaSt were investigated as main factors, a total of eight experimental compounds were obtained. Compound **1** contained no additives and was therefore not processable. In Compound **2**, only I168 was dosed, while Compound **3** contained only T622. Compound **4** was compounded with both I168 and T622. Compound **5** comprised only CaSt, whereas Compound **6** consisted of I168 and CaSt. Compound **7** was formulated with T622 and CaSt, and Compound **8** contained all three additives. These predefined ratios and concentrations formed the basis for the calculations in the DoE evaluation. After each compounding step involving a change of additives, the dosing unit was cleaned.

The extruder operated at a total output of 20 kg/h during the production of the test formulations. The barrel temperature at the feed zone was set to 40 °C and progressively increased from 190 °C to 220 °C across the extrusion zones. After exiting the nozzle, the extrudate was conveyed through a water bath, subsequently dried, and finally pelletized using cold cutting.

### 2.3. Analysis of Additive Contents

The evaluation and interpretation of the DOE are based on the additive concentrations as introduced. During compounding and the subsequent processing steps, partial degradation of the additives, however, occurs due to their active participation in the reaction and stabilization mechanisms. Therefore, additive contents were determined immediately after compounding.

#### 2.3.1. GC-MS

To determine the T622 content, a sample weight of 1 g was measured. The sample was then boiled in 25 mL toluene (Fisher Scientific GmbH, Schwerte, Germany) for 30 min while stirring and reflux cooling until the sample was dissolved. After dissolution, 50 mL of a 0.1 M KOH (Fluka AG, Buchs, Switzerland) solution in methanol (Fisher Scientific GmbH, Schwerte, Germany) was added as a derivatizing agent and was boiled for a further 15 min with stirring and reflux cooling. According to W. Freitag and R. Wurster, the saponification of T622 to the corresponding 1-(2-Hydroxyethyl)-2,2,6,6-tetramethylpiperidin-4-ol (Diol 622) was performed [15]. M. A. Jimenez et al. compared the use of KOH and TBAH and recommended the use of KOH due to its high speed and efficiency [16]. In addition to the derivatization of T622, the PP-C precipitated from the solution due to the addition of methanol. After boiling, the extract was cooled for 15 min using reflux cooling. The entire extract was then poured off the precipitated polymer and filtered using a 20 µm syringe filter. At a water bath temperature of 60 °C, the filtered extract was evaporated to dryness in a rotary evaporator operating under vacuum. The residue was dissolved in 25 mL of methanol and filtered again using a 20 µm syringe filter.

The GC/MS Clarus 600/SQ8 GC/MS Spectrometer (GC/MS) (PerkinElmer, Waltham, MA, USA) was used for the gas chromatographic analysis. To measure the T622 content, 1 µL of the generated extract was injected via the 250 °C injector. The GC column used, Elite 5 MS (30 m, ID 0.25 mm, 0.25 μm), was tempered to 100 °C at the start of the measurement. This temperature was kept isothermal for 1 min and then heated up to 250 °C at 10 °C/min. The helium carrier gas used ran at a flow rate of 1 mL/min. The measurement was performed spitless. The detection was carried out using selective ion monitoring (SIM) of the masses 102, 170 and 186 *m*/*z*.

Quantification was performed by external standard calibration using Diol 622 (abcr GmbH, Karlsruhe, Germany), dissolved in methanol. The dilution series for calibration comprised concentrations of 10–90 µg/mL Diol 622, which corresponds to a Diol 622 content of 0.03–0.3% by weight. The sample concentration was calculated using the linear regression of the peak area as a function of the known concentration. To derive the T622 from the Diol 622, the concentration was multiplied by a factor of 1.41 [15].

#### 2.3.2. LC-DAD

To determine the concentration of I168, 2 g of sample was boiled in 20 mL toluene (Fisher Scientific GmbH, Schwerte, Germany) for 45 min at 115 °C whilst cooling under reflux. After the PP had dissolved, 25 mL of methanol (Fisher Scientific GmbH, Schwerte, Germany) was added. The PP precipitates due to its insolubility in methanol, and I168 remains in the solution. The resulting extract was made up to 50 mL with a 1:1 mixture of methanol (Fisher Scientific GmbH, Schwerte, Germany) and toluene (Fisher Scientific GmbH, Schwerte, Germany). The extract was then filtered using a syringe filter with a pore size of 20 µm and diluted 1:1 with methanol (Fisher Scientific GmbH, Schwerte, Germany).

The HPLC Agilent 1260, including a DAD detector (Agilent Technologies, Santa Clara, CA, USA), was used for the liquid chromatographic analysis. The column used is the PerfectChrom 100 C18M 5 µm 125 × 4.6 mm HPLC Column (MZ-Analysentechnik, Mainz, Germany). To determine the I168 content, a gradient of two solvent mixtures was used. Mobile phase A consists of 80 vol.% methanol (Fisher Scientific GmbH, Schwerte, Germany) and 20 vol.% bidistilled water, phase B consists of 50 vol.% ethyl acetate (Fisher Scientific GmbH, Schwerte, Germany) and 50 vol.% acetonitrile (Fisher Scientific GmbH, Schwerte, Germany). The system was equilibrated to 50% mobile phase A and 50% mobile phase B. Within 12 min, a gradient proceeded until 100% mobile phase B was set. This state was maintained for 5 min. The measurement was performed at a flow rate of 0.8 mL/min. The column oven was heated to 25 °C, and the DAD detector was set to 272 nm. The measurement was based on the proposed method of the Bundesanstalt für Materialforschung und- prüfung for the determination of the type and concentration of phenolic and phosphitic antioxidants in sealing strips made of high-density polyethylene (PEHD) [17].

An external standard calibration with Tris(2,4-di-tert-butylphenyl)phosphite (I168) supplied by Merck KGaA (Darmstadt, Germany) was carried out for quantification. The standard was dissolved in a 1:1 mixture of toluene and methanol (each from Fisher Scientific GmbH, Schwerte, Germany). The calibration series includes an I168 standard concentration of 6–42 µg/mL, which correlates with an additive concentration in the sample of 0.016–0.1 wt.%. To calculate the concentration of I168 in the sample, the measuring points of the calibration series are calibrated as a function of the known concentrations.

#### 2.3.3. Complexometric Titration

The test specimens were cut, and a mass of 10 g was placed in porcelain crucibles. The crucibles containing the samples were subsequently heated in an oven at 850 °C for 20 min. After cooling to room temperature, the residues were dissolved in 10 mL of 1 M nitric acid (Merck KGaA, Darmstadt, Germany). The resulting solutions were then diluted with bidistilled water to a final volume of 100 mL.

Aliquots of 20 mL from these solutions were further diluted to 100 mL with bidistilled water. Following the addition of an indicator buffer tablet (Merck KGaA, Darmstadt, Germany) and 1 mL of concentrated ammonium hydroxide solution (Merck KGaA, Darmstadt, Germany), the solutions were titrated with 0.0002 M Titriplex III solution (Merck KGaA, Darmstadt, Germany) until the color transitioned from red to green. The calcium mass fraction in the PP-C formulations was calculated using Equation (1).


(1) $$wt.\%\;Ca=\frac{c\times V\times M\times 100}{20\times m_{PP-C}}\times 100\%$$

*wt.%* Ca: Mass fraction of calcium in the PP-C formulation [%]

*c*: Concentration of Titriplex III

*V*: Determined titration volume

*M*: Molar mass of calcium

*m*_PP−C_: Mass of PP-C

### 2.4. Preparation of Test Specimens

Type 5A tensile test specimens were produced in accordance with DIN EN ISO 527-2-06 [18]. via injection molding using a Babyplast 6/10 P device (Rambaldi srl, Molteno Lecco, Italy). The polymer melt (metering stroke: 31.3 mm) was injected at an injection pressure of 55 bar. At the 2 mm switchover point, a holding pressure of 50 bar was applied for 4 s. The cylinder temperature was set to 200 °C, and the mold was tempered by 20 °C. A remaining cooling time of 15 s was maintained. Once a processing-testing cycle was completed, the next cycle was initiated, which included grinding the test specimens and subsequently reprocessing them in the injection molding process.

The samples were processed in three consecutive cycles. In the following, cycles 1 and 3 were compared according to the test plan. Depending on the DOE run, some samples were irradiated with γ-irradiation after each processing cycle. The γ-irradiation was conducted under ambient conditions by BGS GmbH (Wiehl, Germany), with a target dose of 25 kGy and a dose rate of 6 kGy/h. This resulted in samples produced up to three times without the influence of γ-irradiation and samples treated by the influence of γ-irradiation.

All samples were analyzed over a defined period of 4 weeks following either processing or γ-irradiation to minimize the influence of aging effects.

### 2.5. Statistical Experimental Design

A full factorial experimental design is applied. The experimental effort is calculated using Equation (2) [13].


(2) $$n_{r}=n_{l}^{n_{f}}$$

*n_r_*: Experimental effort

*n_l_*: Number of factor levels

*n_f_*: Number of Factors

The factors in the coded form analyzed in this study are presented in Table 2. In the entire experimental design, two factor levels were considered, which were coded as 1/−1. The settings of the influencing factors for the respective levels can also be seen in Table 2. This results in 32 combinations.

The focus of the study was the evaluation of the recyclability of PP in general and under the aspect of sterilization in medical technology. For this reason, the cycle effects A: Processing and B: Irradiation (γ-irradiation) were selected. Furthermore, the influences of the various additives were to be evaluated. A standard stabilization package from medical technology was chosen. The additive quantities added during compounding are used for interpretation (see Table 2). The concentrations measured after compounding in accordance with Section 2.3 are shown in Table 3.

The main effects and their interactions can be used to quantify the effect of the respective factor on the response. The mean values of the responses (quality characteristics) were evaluated. In a linear description model, the influencing factors were then calculated for the response according to Equation (3) [13].


(3) $$y=c_{0}+\sum_{i=1}^{n_{f}}c_{i}x_{i}+\sum_{i=1}^{n_{f-1}}\sum_{j=i+1}^{n_{f}}c_{ij}x_{i}x_{j}+\varepsilon$$

y: Response

c_0_ … c_23_: Modell coefficients

n_f_: Number of Factors

xi,j (oder 1 … 5): Input variables

ε: Residual error

Above all, the formulation of the material had an influence on its processability over repeated cycles. Individual runs could not be carried out due to the lack of processability and were therefore ignored in the evaluation (Runs 1–3, Run 12). The YI, the crystallization temperature, the elongation at break and the zero-shear viscosity were evaluated as the responses.

The linear fit was suitable for calculating the models of all responses; no curvature was detected. Design-Expert Software Stat-Ease 360 (Stat-Ease, Inc., Minneapolis, MN, USA) was used for statistical evaluation. The ANOVA and the R^2^ values are presented in the following to illustrate the statistical results of the responses. The models are considered significant when the condition *p* < 0.05 is met. The artwork was created using Design-Expert Software (Stat-Ease, Inc., Minneapolis, MN, USA) and OriginPro 9.0 (OriginLab, Northampton, MA, USA).

### 2.6. Responses

#### 2.6.1. YI Determination

The degree of yellowing was assessed using the YI in accordance with DIN 6167 [19]. The CIE (International Commission on Illumination) color space, defined as XYZ, was applied in accordance with DIN 5033 [20]. YIs were measured using a CM-5 spectrophotometer (Konica Minolta, Marunouchi, Japan) with a 10° observer angle and standard daylight type D65. For measuring, an aperture with a diameter of 3 mm was used, onto which the specimens were centrally positioned.

#### 2.6.2. Differential Scanning Calorimetry

The Differential Scanning Calorimetry (DSC) measurements were conducted with the DSC822e from Mettler Toledo (Greifensee, Switzerland). The MX5 balance from Mettler Toledo (Greifensee, Switzerland) was used to determine the sample weight. The sample with a mass of 10 mg was positioned in an aluminum crucible (40 µL) and closed with a pierced lid. The measurement ranged from 25 to 200 °C with a heating or cooling rate of 10 K/min. Two heating and one cooling ramps were measured. The results of the cooling process were used to evaluate the statistical test plan. The STARe V16.30a software from Mettler Toledo (Greifensee, Switzerland) was used for setting and subsequent evaluation.

#### 2.6.3. Tensile Tests

The tensile tests were executed with zwickiLine Z2.5 from Zwick Roell (Ulm, Germany) in accordance with DIN EN ISO 527-1 [21]. The initial load was 0.1 MPa. Measurements were taken at a measuring speed of 50 mm/min. The measuring speed for determining the E-Modulus was 1 mm/min. The manufactured 5A test specimens were measured. In the first step, the thickness and width of the samples were measured using an electronic caliper gauge. The samples were clamped in the center of the clamps at a distance of 40 mm. The sample was measured until failure. The measurement control and evaluation were carried out using testXpert II from Zwick Roell (Ulm, Germany).

#### 2.6.4. Oscillatory Rheological Measurement

The oscillation rheometric measurements were carried out with the ARES Rheometric Scientific from TA Instruments (Hüllhorst, Germany) in a plate-plate setup. The diameter of the plates used was 25 mm. The tensile test specimens were used for the measurements. The sample was positioned on the plates and then heated to 180 °C in the oven. After melting the sample, the target gap dimension of 1 mm was approached, and the excess melt was removed. The material was then heated once to 210 °C for 2 min to ensure complete melting of the material. The furnace temperature was then lowered to 180 °C. In order to ensure a uniform temperature of 180 °C over the entire sample, the measurement was performed after 5 min. It was measured in a frequency sweep at 180 °C with a deformation of 20% from 0.4 to 400 rad/s. To ensure that the measurements were carried out in the linear viscoelastic (LVE) range, an amplitude sweep was performed prior to the measurements.

## 3. Results and Discussion

### 3.1. Additive Concentrations Measured Following Compounding

As outlined above, the evaluation of the DOE is based on the additive contents as initially added during compounding. Since the additives perform their stabilizing function and undergo degradation during compounding and subsequent processing steps, their concentrations change in the actual process. The measured additive contents after compounding, in accordance with Section 2.3, are listed in Table 3.

The additive concentrations of the compounds differed according to the respective formulations. The I168 content in formulations without CaSt was only slightly above the limit of quantification. When calcium stearate was present, a higher amount of I168 remained after compounding. Polyolefins polymerized via coordinative polymerization, as the here investigated PP-C, inevitably contain acidic catalyst residues. CaSt acts as an acid scavenger and neutralizes these acidic species. Acidic residues promote degradation of I168 either through direct reactions with the stabilizers or by enhancing the formation of radicals and hydroperoxides [22,23]. In formulations containing CaSt, the concentration of I168 was comparatively high. The presence of T622 slightly reduced the I168 content. This reduction may have originated from processing-related fluctuations and minor analytical deviations. However, an antagonistic interaction between T622 and I168 has been reported in the literature by I. Bauer et al. [24]. They describe a mechanism where the HALS released nitroxyl radicals that scavenged peroxy radicals but were also able to react with the phosphite molecule, thereby consuming Irgafos 168 before it could decompose hydroperoxides. This side reaction removed the active phosphorus center of the phosphite and consequently shortened its effective lifetime.

The T622 content differed among the various compounds; however, no trend was evident. In a previous study, we investigated the behavior of T622 over four recycling cycles with and without γ-irradiation, and similarly, no trend was observed [25]. These fluctuations could be attributed to the reaction mechanism of HALS. Müller and Meier reported that nitroxyl radicals generated from the HALS reacted with alkyl radicals formed during the photoinitiated degradation of PP-C, yielding hydroxylamine ethers. As described by the “Denisov cycle,” these hydroxylamine ethers subsequently reacted with peroxy radicals to form dialkyl peroxides, regenerating the nitroxyl radicals [26]. The calcium stearate content after extrusion was determined via complexometric titration of calcium. No remarkable differences to the initially added amount and between compounds were observed.

### 3.2. Influence of the Main Factors on the Yellowness Index of the PP-Compounds

**Figure 1:**
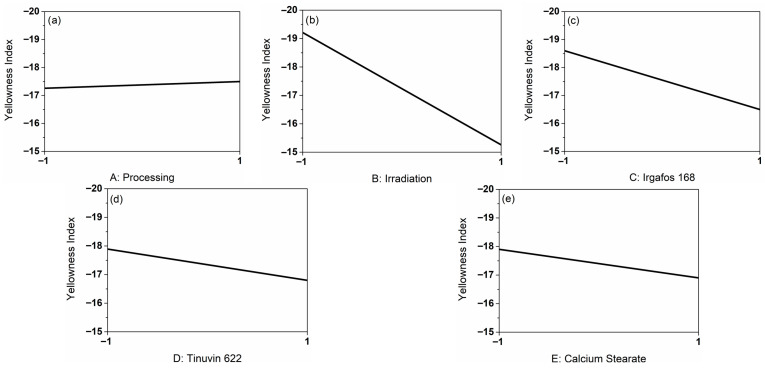
Graphical representation of the main factors on the response YI based on the real measurement results (**a**) shows the influence of processing, (**b**) the influence of irradiation, (**c**) the influence of the additive I168, (**d**) the influence of the additive T622 and (**e**) the influence of the additive CaSt.

To evaluate the optical change, the samples were examined with regard to the YI in function of repeated processing, irradiation and the presence of the additives I168, T622 and CaSt. Figure 1 displays the corresponding line graphs derived from the DoE representing the influencing factors in coded form. These graphs illustrate the effect of each individual main factor on the response variable YI. Specifically, they indicate whether YI increases or decreases depending on the level of a given factor, e.g., whether irradiation was applied, independent of the other factors. Thus, line graphs provide an initial qualitative assessment of the influence of the investigated factors on the response.

Graph (a) shows the influence of processing on the YI. In cycle 1 (coded −1), the YI is 0.24 lower than in cycle 3 (coded 1). Since the slope of the graph with 0.12 is very small, the influence of processing was minimal. When considering graph (b), it is noticeable that the influence of irradiation was greater, since the slope of 1.98 is larger. Overall, the effect of the additives (c–e) was minor. The YI decreased by ΔYI = 2.1 in the presence of I168, by ΔYI = 1.09 in the presence of T622, and by ΔYI = 1.0 in the presence of CaSt. When evaluating the main effects, it became apparent that the influence of irradiation with 25 kGy γ-radiation on the YI was greatest. Negative YI values correspond to samples exhibiting very low or no yellow discoloration. An increase in YI from −19 to −15 induced by irradiation is not perceptible to the human eye.

Pareto charts, which show the identified influencing factors and interactions sorted by size, are particularly suitable for evaluating the influences on defined responses [13]. In the Pareto chart showing the influences on the YI (Figure S1, Supporting Information), we observe a clear distinction of the irradiation influence. Because of this, only factor B: Irradiation was used for the model calculation. To create a prediction model from the experimental design, the main effects were selected. Effects of a smaller influence were ignored. The determined model is represented by Equation (4) (coded).


(4) $$YI=-17.31+1.99\times F_{Irradiation}$$

*YI*: Yellowness Index

*F_Irradiation_*: Influencing Factor Irradiation

Both the adjusted R^2^ of 0.23 and the predicted R^2^ of 0.14 are very low. Since the influence of the main factors is so small, it is not possible to generate a meaningful prediction model using the factors considered. Overall, the measured yellowing indices (YI) were very low, indicating minimal discoloration. The observed yellowing was below the threshold of human visual perception, consistent with previously reported values for similarly stabilized polyolefins [27].

For illustration, the differences in the change of the YI depending on the irradiation for selected runs of cycle 1 and 3 are shown in Table 4. The main factors (C–E) correspond to level 1, thus containing all three additives I168, T622 and CaSt. The influence of irradiation was clearly visible in an increase in YI, whereas the influence of processing without irradiation had no effect on yellowing.

Both γ-irradiation and processing result in the formation of radicals. The subsequent auto-oxidation occurs, and functional groups are formed, which may be associated with the change in color of the material [26,28]. In the literature, the formation of chromophore groups and the resulting yellowing are attributed to the degradation of phenolic and phosphitic antioxidants [8,26]. In a previous study, a significantly higher discoloration could be measured as visible by a positive YI after processing and irradiation. Due to the unknown stabilizing formulation used in that study, it can be assumed that phenolic antioxidants were used [27]. In the present study, the compound does not contain any phenolic antioxidants, which can thus not lead to an increase in the YI. Due to the much stronger influence of irradiation on yellowing than of processing, it can be assumed that radicals are more likely to be formed by irradiation than by injection molding.

### 3.3. Influence of the Main Factors on the Crystallization Temperature of the PP-Compounds

To evaluate the crystallization behavior of the PP-C, the crystallization temperature was considered.

**Figure 2:**
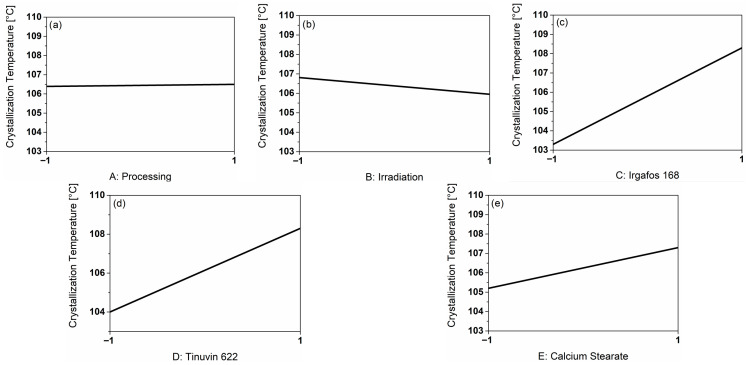
Graphical representation of the main factors on the response crystallization temperature [°C] based on the real measurement results. (**a**) shows the influence of processing, (**b**) the influence of irradiation, (**c**) the influence of the additive I168, (**d**) the influence of the additive T622 and (**e**) the influence of the additive CaSt.

Figure 2a shows the influence of processing, which resulted in a slight increase of 0.1 °C in the crystallization temperature after cycle 3 (coded 1). In (b), the effect was slightly greater, with a decrease of 0.9 °C due to irradiation. The influence of the additives (c–e) resulted in an increased crystallization temperature. In the presence of I168, the crystallization temperature increased by 5.0 °C; in the presence of T622 by 4.3 °C; and in the presence of CaSt by 2.1 °C. The effect of CaSt addition was the smallest.

The Pareto chart of the crystallization temperature can be found in Figure S2 in the Supporting Information. The interactions of the additives I168+CaSt, I168+T622 and T622+CaSt had the greatest effect and stood out clearly from the other influences. To generate a model, it is important to consider the main factors used and the relevant interactions [29]. From these conditions and observations, the model in Equation (5) (coded) resulted.


(5) $$T_{C}=108+0.34F_{I168}+0.05F_{T622}-0.79F_{CaSt}+3.68F_{I168}F_{T622}+\;3.88F_{I168}F_{CaSt}+3.68F_{T622}F_{CaSt}$$

*T_c_*: Crystallization Temperature [°C]

*F*_*I*168_: Factor I168

*F*_*T*622_: Factor T622

*F*_*CaSt*_: Factor CaSt

The resulting model is evaluated by discussing the Table 5.

The conditions for the significance of the model are fulfilled. The predicted and adjusted R-squares are very high and fulfill the condition of a difference of less than 0.2. With this model, the crystallization temperature can be predicted with a probability of 94%.

**Figure 3:**
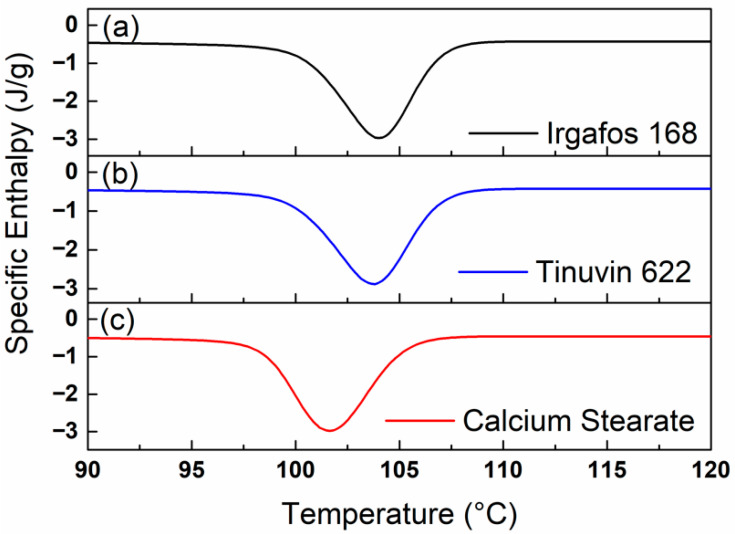
Zoom in thermograms (region of crystallization) of the compounds containing either I168 (**a**), T622 (**b**) or CaSt (**c**). Curves represent the registration of the first cooling run in DSC measurements. Main factors A: Processing and B: Irradiation correspond to −1, the measured values resulted from the non-irradiated cycle 1.

Figure 3 shows thermograms of PP compounds with either I168, T622 or CaSt as a zoom on the crystallization event. The curves exhibit identical profiles. However, the peak maximum of the sample containing CaSt is shifted toward lower temperatures. The difference between the crystallization temperatures of the I168 or the T622 compound is approximately 3 °C compared to the CaSt compound. The lower the crystallization temperature, the shorter the molecular chains, which indicates a breakdown of the molecular chains [30,31]. Therefore, we suppose that observed differences primarily result from variations in chain length and the formation of functional groups, which are consequences of material degradation. Processing in the extruder, but also injection molding, insduce thermooxidative degradation, which could be suppressed more or less depending on the stabilizer package. As a result of these measurements, CaSt provided less protection for the polymer against material degradation. CaSt is an acid scavenger and is therefore particularly relevant immediately after polymerization to neutralize acids that have formed. Radicals formed during subsequent compounding and processing can be captured by radical scavengers and hydroperoxide decomposers such as T622 and I168, which is evident in improved stabilization [26].

### 3.4. Influence of the Main Factors on the Elongation at Break of the PP-Compounds

**Figure 4:**
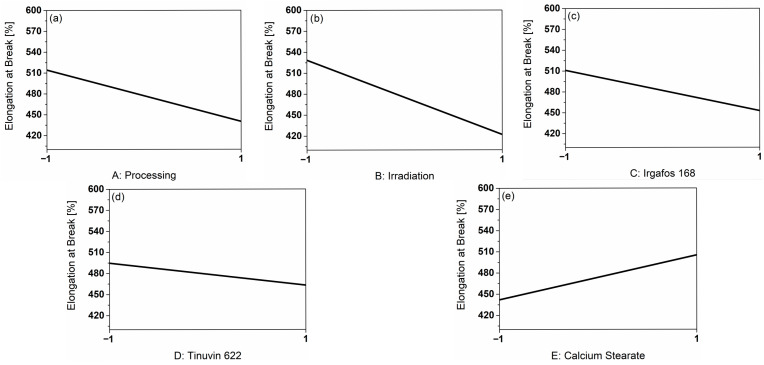
Graphical representation of the main factors on the response elongation at break [%] based on the real measurement results. (**a**) shows the influence of processing, (**b**) the influence of irradiation, (**c**) the influence of the additive I168, (**d**) the influence of the additive T622 and (**e**) the influence of the additive CaSt.

To evaluate the factors influencing the mechanical properties, the response of elongation at break was considered. The influences of the main factors on the elongation at break based on the real measurement results are shown in Figure 4.

Both A: Processing (Figure 4a) and B: Irradiation (Figure 4b) induced a decrease in elongation at break. Repeated processing led to a decrease in elongation at break of 74.2%, while irradiation resulted in a reduction of 106.6%. The influences of the additives (c–e) had a minor influence on the elongation at break. In the presence of I168, the elongation at break was lower by 58.3%, by 31.6% in the presence of T622, and by 63.2% in the presence of CaSt. The presence of the additive CaSt in Figure 4e had the greatest influence among the additives.

Resulting from the Pareto chart of the elongation at break in Figure S3 in the Supporting Information, the factors A: processing and B: irradiation had the greatest effect on elongation at break and resulted in its decrease. These two factors stood out and were therefore used to calculate the model presented in Equation (6) (coded).


(6) $$\varepsilon_{B}=474.90-40.44F_{Processing}-54.08F_{Irradiation}$$

*ε_B_*: Elongation at Break [%]

*F_Processing_*: Factor Processing

*F_Irradiation_*: Factor Irradiation

The Adjusted R^2^ of 0.43 and the Predicted R^2^ of 0.34 of the model are not convincing. The influence of the factors considered does not sufficiently stand out from the signal-to-noise ratio.

**Figure 5:**
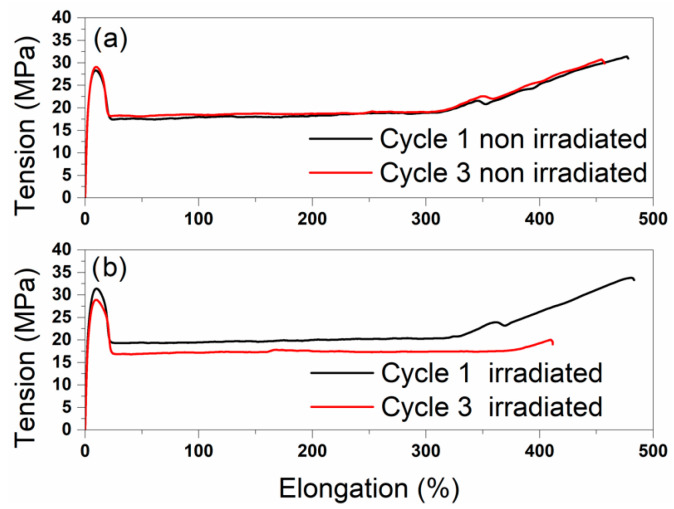
Stress–strain diagrams after 1 and 3 processing cycles. The results for B = −1 (non-irradiated) are represented in (**a**), and the results for B = 1 (irradiated) in (**b**). Further, the main factors C–E equal 1, thus the presence of the three additives I168, T622 and CaSt.

Figure 5 shows corresponding stress–strain diagrams depending on the main factors, A: Processing and B: Irradiation. The elongation at break in % can be identified as the endpoint of the measurement curve. The elongation at break decreased after three cycles, both with and without the influence of γ—irradiation. Without irradiation, the elongation at break decreased only slightly over the processing cycles (20.9%), whereas the comparison of the irradiated samples from cycles 1 and 3 showed a reduction of 71.7%. Under the influence of γ—irradiation, the decrease was more pronounced. The decrease in elongation at break is a well-known indicator of material degradation due to chain scission [14,32]. T. Kremser et al. pointed out a significant drop in elongation at break after falling below a critical molar mass [8]. The low predictability of the model suggests that the influence on the response elongation at break was low. As a result, it can be assumed that the critical molar mass was not exceeded in this study. After three processing cycles under the influence of irradiation, the elongation at break was still high at approximately 400% and makes noticeable embrittlement unlikely [8]. The literature describes increased embrittlement when stabilizers are absent [27]. Figure 4 shows an increase in elongation at break with regard to the presence of CaSt. When CaSt reacts with acids, such as hydrochloric acid resulting from coordinative polymerization, calcium chloride (CaCl_2_) and stearic acid are formed. Stearic acid is commonly used in polymer modification as a processing aid and lubricant. It aligns between polymer chains, reduces van der Waals interactions, and thereby softens the material. In the present study, this effect manifested in an increase in the elongation at break of the polymer [26]. The influence of the other two additives was not as pronounced under the conditions considered (Figure S3, Supporting Information).

### 3.5. Influence of the Main Factors on the Zero Shear Viscosity of the PP-Compounds

**Figure 6:**
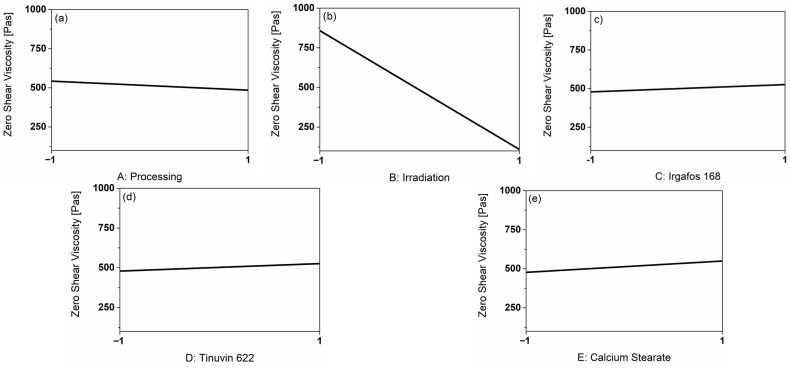
Graphical representation of the main factors on the response zero shear viscosity [Pas] based on the real measurement results. (**a**) shows the influence of processing, (**b**) the influence of irradiation, (**c**) the influence of the additive I168, (**d**) the influence of the additive T622 and (**e**) the influence of the additive CaSt.

The evaluation of the zero-shear viscosity was chosen to assess the rheological properties. The influences of the main factors based on the real measurement results are shown in Figure 6.

When comparing the effects of the main factors on the zero-shear viscosity in Pas, it is very noticeable that the factor B: Irradiation in particular reduces the zero-shear viscosity by 747.9 Pas. The presence of additives (see Figure 6c–e) resulted in a slight increase in zero-shear viscosity. The presence of I168 resulted in an increase in the zero-shear viscosity by 48 Pas, whereas CaSt led to a higher characteristic value by 73 Pas. The influence of processing was very small, with a decrease of 57.4 Pas after three processing steps (coded 1), especially compared to the influence of irradiation. However, multiple processing still resulted in a slight decrease in the zero-shear viscosity in the absence of irradiation.

As shown in the Pareto chart of the zero shear viscosity in Figure S4 in the Supporting Information, the influence of irradiation had a decisive influence and exceeded the influence of all other main factors and interactions by far. The presence of the additives only provoked a slightly higher zero-shear viscosity; the radiation-induced degradation could not be suppressed effectively.

For this reason, only the main factor of irradiation was considered in the model calculation. The resulting model is shown in Equation (7) (coded).


(7) $$\eta_{0}=497.91-373.63F_{Irradiation}$$

*η*_0_: Zero shear viscosity [Pas]

*F_Irradiation_*: Factor Irradiation

The statistical evaluation is summarized in Table 6.

Both the F-value and the *p*-value classify the model as significant. The adjusted and predicted R^2^ fulfill the condition of a difference of <0.2 and thus show reasonable agreement. With the determined model, the prediction probability is 81%.

**Figure 7:**
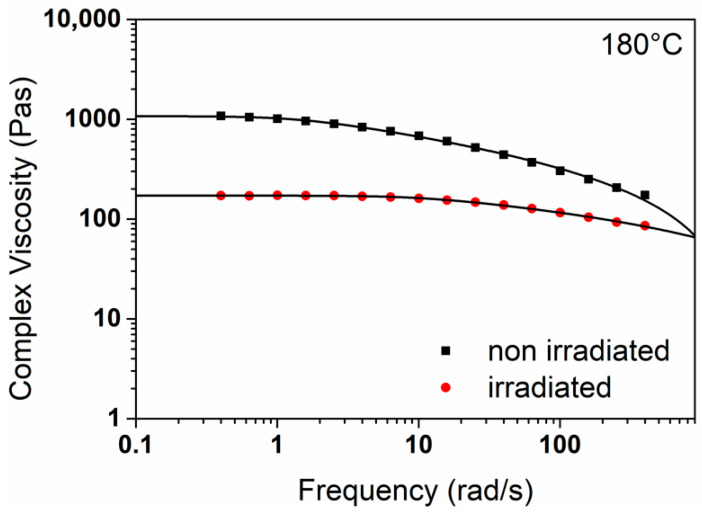
Complex viscosity [Pas] as a function of the factor irradiation (set to −1 and 1) measured at 180 °C. The lines show the evaluation of the measurement data based on the Carreau model [33]. The axes are shown logarithmically. The main factor A equal −1 (Cycle 1), whereas the main factors C–E equal 1. All three additives were present in the formulation.

The influence of B: Irradiation is illustrated in Figure 7.

Figure 7 shows the complex viscosity in Pas as a function of the frequency in rad/s. The black rectangles can be assigned to the non-irradiated sample from cycle 1; the curve shifts to the results of the red circles after the influence of the irradiation. In the formulation considered, all additives, I168, T622 and CaSt were present. Complex viscosity and the resulting zero shear viscosity decreased by 84% due to irradiation. The graph trends remained comparable. There is a direct correlation between the viscosity and the molar mass of a polymer. If the molar mass decreases as a result of chain shortening, a decrease in viscosity, represented here by zero shear viscosity (complex viscosity at f = 0.1 rad/s), is to be expected [34,35,36]. Both irradiation and multiple processing can result in material degradation, which should be suppressed by the stabilizer package used [26]. From this, it can be deduced that material degradation resulted primarily from irradiation.

## 4. Conclusions

The aim of the study was to evaluate the influence of selected factors on a PP copolymer. The main factors considered were the process-related influences of multiple injection molding and sterilization using 25 kGy γ-irradiation and the formulation-influencing factors of the presence of I168, T622 and CaSt. The choice of process and additives resulted from the application in medical technology, but it is also applicable to many other technological fields. The influence of these process and formulation factors was to be analyzed on defined responses. As response values, important material properties with regard to optics (YI), thermal properties (crystallization behavior), mechanics (elongation at break), as well as rheology (zero shear viscosity) were evaluated. The design and evaluation of the study were carried out using the standardized procedure of statistical experimental design. In order to gather as much information as possible, the experimental plan was applied as a full factorial plan. This enabled the determination of a prediction model for future calculations, the statistical evaluation of the results in their entirety and the evaluation of the factors and their interactions using a Pareto chart.

The optical properties were determined using the YI according to DIN 5033. Resulting from these measurements, irradiation had the greatest influence on the yellowing of the compounds, with an increase in YI of ~4. However, the discoloration of the test specimens was rather small and did not stand out from the signal-to-noise ratio. No visually detectable material discoloration was observed. The yellowing of the material due to the addition of the additives in the concentrations given was small. The overall model was, due to the slight changes, rather weak (predicted R^2^ of only 14%).

The crystallization temperature was discussed to assess influences on the crystallization behavior. The crystallization temperature was primarily influenced by the interactions of the additive formulation. The presence of I168 resulted in a ~5 °C higher crystallization temperature. When T622 is present, a ~4 °C higher crystallization temperature was measurable, and with the presence of CaSt, the crystallization temperature increased by ~2 °C compared to compounds without CaSt. The presence of the additives significantly influenced the crystallization temperature and allowed an accurate prediction for future experiments.

The elongation at break was the selected parameter for evaluating the mechanical properties. It was significantly influenced by the process factors of processing and irradiation. The prediction probability of the model with 34% shows that the factors considered did not stand out from the signal-to-noise ratio, and the elongation at break remains stable under the conditions considered. The study fundamentally confirms the assumption that elongation at break was a suitable indicator of material degradation due to chain shortening [7].

The last response considered was the effect of the selected factors on complex viscosity and zero shear viscosity. Zero shear viscosity is a reliable value for evaluating molecular weight due to its correlation. The zero-shear viscosity was primarily influenced by the γ-irradiation. This effect superimposed all other effects and interactions. The resulting prediction probability of 81%, resulting from a model that only takes irradiation into account, confirms this assumption. The effect on the zero-shear viscosity was significantly greater than on all other material properties considered. This characteristic value was very sensitive with regard to chain scission. Since the zero-shear viscosity has dropped significantly by 84%, but the mechanical properties and primarily the elongation at break, have remained at a high level, it can be assumed that chain scission has occurred, but that it has not dropped below a critical level. Otherwise, a more pronounced embrittlement and thus a significantly lower elongation at break would have been observed [7].

In conclusion, the irradiation had the greatest influence on the material properties compared to all other factors considered. This study demonstrates that the applied additives were not able to suppress radiation-induced degradation. To further improve radiation stability in future work, polymer modification approaches may be considered, such as reducing the degree of crystallinity [8] or selectively adding stabilizing additives after specific processing steps. To evaluate material degradation due to chain shortening, the evaluation of zero shear viscosity is primarily suitable. However, in order to be able to subsequently evaluate the effects of the application, the elongation at break should always be considered in combination. This study showed that, despite significantly reduced viscosity and the associated reduced molar mass, good mechanical properties could be achieved. The factors considered indicate that even after three processing and irradiation cycles, a high-quality polymer remains, which is capable of being recycled.

## Figures and Tables

**Table 1:** Additive concentration setting for extrusion.

Additive	Set Concentration [wt.%]
I168	0.15
T622	0.18
CaSt	0.05

**Table 2:** Assignment of influencing factors in coded form.

Influencing Factors in Coded Form		−1	1
A	Processing Cycle	1	3
B	Irradiation [kGy]	0	25
C	I168 Content [wt.%] *	0	0.15
D	T622 Content [wt.%] *	0	0.18
E	CaSt Content [wt.%] *	0	0.05

* as added during compounding.

**Table 3:** Mean value and standard deviation of the additive concentrations in compounds after extrusion, *n* = 3 measurements per compound (Limits of quantification of I168 0.0001 wt.% and of T622 0.0075 wt.%).

Compound	Irgafos 168 [wt.%]	Tinuvin 622 [wt.%]	Calcium Stearate [wt.%]
**1**	0	0	0
**2**	0.0015 ± 0.0001	0	0
**3**	0	0.1651 ± 0.0018	0
**4**	0.0007 ± 0.0001	0.1800 ± 0.0045	0
**5**	0	0	0.050 ± 0.005
**6**	0.0299 ± 0.0003	0	0.052 ± 0.005
**7**	0	0.1841 ± 0.0025	0.055 ± 0.005
**8**	0.0245 ± 0.0022	0.1699 ± 0.0053	0.045 ± 0.005

**Table 4:** Results of the YI for the variation of the main factors A: processing and B: irradiation. The main factors C-E correspond to 1 in the coded form (Table 2), *n* = 5 measurements.

Cycle	YI (DIN6167)	
	Non-Irradiated	Irradiated
1	−15.7 ± 0.6	−11.9 ± 0.6
3	−15.7 ± 0.5	−8.6 ± 0.7

**Table 5:** Results of the statistical analysis of the model and the statistics of the response crystallization temperature.

F-Value	100.85
R^2^	0.97
Adjusted R^2^	0.96
Predicted R^2^	0.94

**Table 6:** Results of the statistical analysis of the model and the fit statistics of the response zero shear viscosity.

F-Value	131.5
R^2^	0.84
Adjusted R^2^	0.83
Predicted R^2^	0.81

## Data Availability

The original contributions presented in this study are included in the article/supplementary material. Further inquiries can be directed to the corresponding authors.

## Supplementary Materials

The following supporting information can be downloaded at: https://www.mdpi.com/article/10.3390/polym18010083/s1, Figure S1: Display of the standardized effect of the main factors and interactions sorted by the size of the influence on the YI; Figure S2: Display of the standardized effect of the main factors and interactions sorted by the size of the influence on the crystallization temperature; Figure S3: Display of the standardized effect of the main factors and interactions sorted by the size of the influence on the elongation at break; Figure S4: Display of the standardized effect of the main factors and interactions sorted by the size of the influence on the zero shear viscosity.

## References

[1] Joseph B., James J., Kalarikkal N., Thomas S. (2021). Recycling of medical plastics. Adv. Ind. Eng. Polym. Res..

[2] Ramos T., Christensen T.B., Oturai N., Syberg K. (2023). Reducing plastic in the operating theatre: Towards a more circular economy for medical products and packaging. J. Clean. Prod..

[3] Beloeil H., Albaladejo P. (2021). Initiatives to broaden safety concerns in anaesthetic practice: The green operating room. Best Pract. Res. Clin. Anaesthesiol..

[4] Pegg M., Rawson R., Okere U. (2022). Operating room waste management: A case study of primary hip operations at a leading National Health Service hospital in the United Kingdom. J. Health Serv. Res. Policy.

[5] Grigoriadi K., Nooijens M., Vanhouttem M.M., Barthelemy V., Klemm B., Boersma A. (2024). The role of recy-cling in UV and thermal ageing of polypropylene block copolymer. Polym. Degrad. Stab..

[6] Hamskog M., Klügel M., Forsström D., Terselius B., Gijsman P. (2004). The effect of base stabilization on the recyclability of polypropylene as studied by multi-cell imaging chemiluminescence and microcalorimetry. Polym. Degrad. Stab..

[7] Thomas K., Markus S., Stefan R., Joachim K., Dirk W. (2019). Schubert, Degradation studies of a commercial radiation-resistant polypropylene sterilized by gamma and electron beam technology before and after subsequent accelerated aging cycles. J. Appl. Polym. Sci..

[8] Kremser T. (2020). *Strahlensterilisation medizinischer* Einmalartikel. Ph.D. Thesis.

[9] Ikram H., Halim H. (2002). Redhwi, Development of polypropylene-based ultraviolet-stabilized formulations for harsh environments. J. Mater. Eng. Perform..

[10] Allen N.S., Edge M., Hussain S. (2022). Perspectives on yellowing in the degradation of polymer materials: In-ter-relationship of structure, mechanisms and modes of stabilization. Polym. Degrad. Stab..

[11] Lisanevich M.S., Galimzyanova R.Y., Rakhmatullina E.P., Khakimullin Y., Musin I.N., Tsareva E.E. (2019). Effect of Processing and Radiation Exposure on the Structure and Properties of Polypropylene. KEM.

[12] Plastics Europe (2023). Plastics—The fast Facts 2023 • Plastics Europe. https://plasticseurope.org/knowledge-hub/plastics-the-fast-facts-2023/.

[13] Siebertz K., van Bebber D., Hochkirchen T. (2017). Statistische Versuchsplanung.

[14] Ehrenstein G.W., Pongratz S. (2007). Beständigkeit von Kunststoffen.

[15] Freitag W., Wurster R. (1988). Determination of the polymeric light stabilizer Tinuvin 622 in polyolefins. J. Chromatogr..

[16] Jiménez M.A., Recio I.P., Chacón L.D., Fermín R.B. (2007). Determi-nation of Tinuvin 622 in polyethylene samples by liquid chromatography with ultraviolet absorption detection. Rev. Téc. Fac. Ing. Univ. Zulia.

[17] Federal Institute for Materials Research and Testing March 2013—Determination of the Type and Concentration of Phenolic and Phosphitic Antioxidants in Sealing Sheets Made of High-Density Polyethylene (HDPE).

[18] (2012). Plastics—Determination of Tensile Properties—Part 2: Test Conditions for Moulding and Extrusion Plastics.

[19] (1980). Description of Yellowness of Near-White or Near-Colourless Materials.

[20] (2017). Colorimetry—Part 1: Basic Terms of Colorimetry.

[21] (2019). Plastics—Determination of Tensile Properties—Part 1: General Principles.

[22] Zhang X.-R., Wang F.-S., Wang X., Gao Y., Zhang H.-X., Liu Z.-Q., Feng J.-C. (2025). Comparative Investiga-tion of the Migration Behavior of Two Stearate Acid Scavengers from Ziegler-Natta Polypropylene into Water during Autoclaving Treatment. Chin. J. Polym. Sci..

[23] Sherman R.L., Kern K.E., Spalding M.A., Chatterjee A.M. (2018). Acid Scavengers for Polyethylene. Handbook of Industrial Polyethylene and Technology: Definitive Guide to Manufacturing, Properties, Processing, Applications and Markets.

[24] Bauer I., Habicher W.D., Korner S., Al-Malaika S. (1997). Antioxidant interaction between organic phosphites and hindered amine light stabilizers: Effects during photoxidation of polypropylene-II. Polym. Degrad. Stab..

[25] Espelage N., Liebal J.L., Behnecke M., Kömpe K., Susoff M.L., Blömer P., Steinhart M., Petersen S. (2025). Exposition of Radiation-Stabilized Polypropylene Copolymer to Repeated Injection Molding and γ -Radiation. J. Appl. Polym. Sci..

[26] Maier R.-D., Schiller M. (2016). Handbuch Kunststoff-Additive.

[27] Zerhusen N., Herrmann S., Susoff M.L., Petersen S. (2024). Stability assessment of a commercial poly-propylene copolymer and Irgafos 168 under repeated injection molding and γ-irradiation cycles. J. Appl. Polym. Sci..

[28] Saha A., Chakraborty V., Chintalapudi S.N. (2000). Chemical modification of polypropylene induced by high energy carbon ions. Nucl. Instrum. Methods Phys. Res. Sect. B Beam Interact. Mater. Andatoms.

[29] Stat Ease, Inc ANOVA. https://www.statease.com/.

[30] Frick A., Stern C. (2013). DSC-Prüfung in der Anwendung.

[31] Meides N., Mauel A., Menzel T., Altstädt V., Ruckdäschel H., Senker J., Strohriegl P. (2022). Quantifying the fragmentation of polypropylene upon exposure to accelerated weathering. Microplastics Nanoplastics.

[32] Williams J.L., Dunn T.S. (1980). Radiation stability of polypropylene. Radiat. Phys. Chem..

[33] Carreau P.J., De Kee D.C.R., Chhabra R.P. (2021). Rheology of Polymeric Systems Principles and Applications.

[34] Auhl D.W. (2006). Molekulare Struktur und Rheologische Eigenschaften Strahlenmodifizierter Polypropylene. Ph.D. Dissertation.

[35] Bonten C. (2016). Kunststofftechnik: Einführung und Grundlagen.

[36] Schröder T. (2018). Rheologie der Kunststoffe: Theorie und Praxis.

