# Supplementary material for: A Design of Experiment (DoE) Approach to Evaluate the Recyclability of a Polypropylene Copolymer in Medical Technology Under the Aspect of Additive Composition
Source: Polymers (Basel). 2025 Dec 27;18(1):83. doi: 10.3390/polym18010083 (PMC12787600; doi:10.3390/polym18010083)
Supplement: Supplementary file 1 [file polymers-18-00083-s001.zip › polymers-4038588-supplementary.pdf]

Supporting information to

“A Design of Experiment (DoE) Approach to Evaluate the Recyclability of a Polypropylene Copolymer in Medical Technology under the Aspect of Additive Composition”

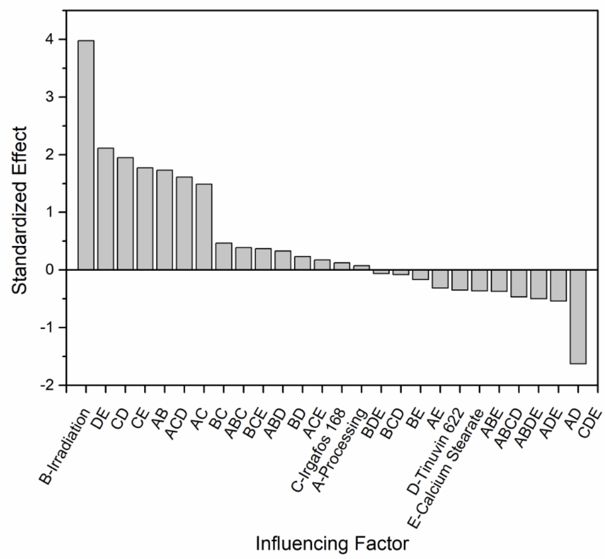

Figure S1: Display of the standardized effect of the main factors and interactions sorted by the size of the influence on the YI

Figure S1 shows the Pareto chart for the response Yellowness Index. Figure S1 clearly shows that B: Irradiation had twice as much influence as all other main factors and interactions. This is followed by the influences of the two-way interactions of the additives. Therefore, only the main factor B:Irradiation is used to create the prediction model.

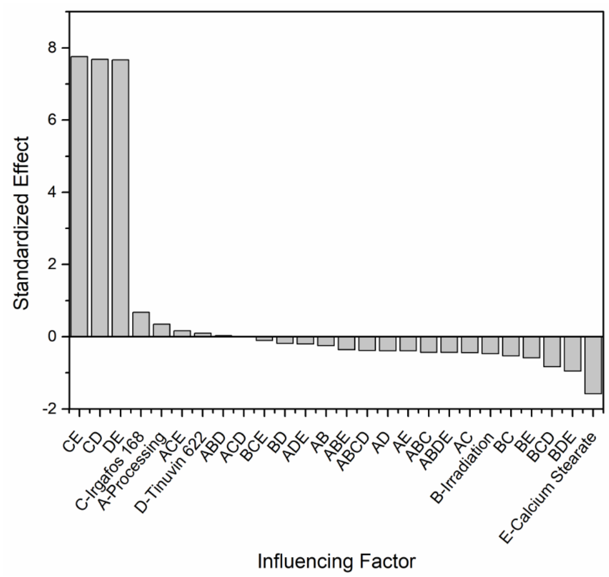

Figure S2: Display of the standardized effect of the main factors and interactions sorted by the size of the influence on the crystallization temperature

Figure S2 shows the Pareto chart for crystallization temperature. The twofold interactions of the additives have a very large influence on crystallization temperature compared to the main factors and the remaining interactions.

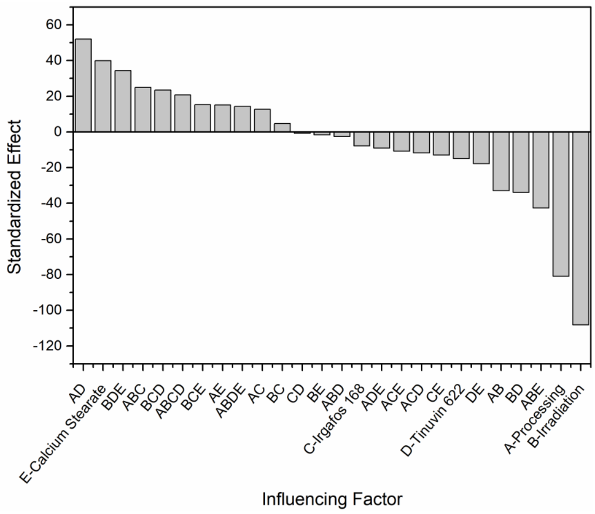

Figure S3: Display of the standardized effect of the main factors and interactions sorted by the size of the influence on the elongation at break

Figure S3 shows the Pareto chart with the influences on elongation at break. The effect of processing and irradiation is greatest and results in a decrease in elongation at break.

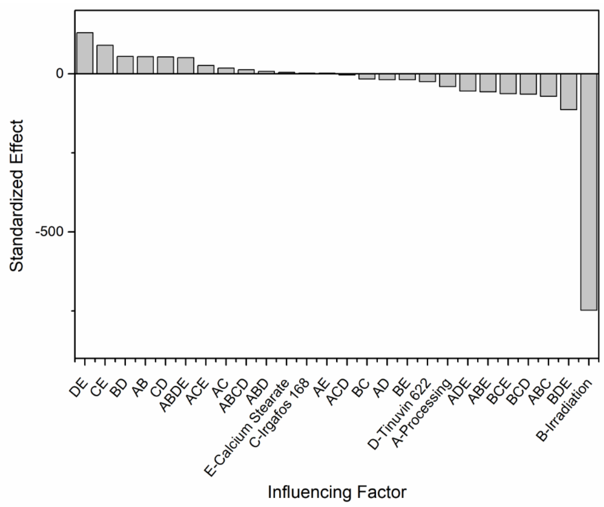

Figure S4: Display of the standardized effect of the main factors and interactions sorted by the size of the influence on the zero shear viscosity

The Pareto chart of zero shear viscosity in Figure S4 shows a clear influence of irradiation on the change in the response, which overshadows all other influences.
